# Nonlinear integration of evidence in a dynamic motor task

**DOI:** 10.1186/1471-2202-12-S1-P148

**Published:** 2011-07-18

**Authors:** Katja Fiedler, J Michael Herrmann

**Affiliations:** 1 MPI for Dynamics and Self-Organization, Bunsenstr. 10, Goettingen, D-37073, Germany; 2 BFNT Goettingen, Bunsenstr. 10, Goettingen, D-37073, Germany; 3 School of Informatics, IPAB & ILSI, University of Edinburgh, 10 Crichton St, EH8 9AB, Scotland, UK

## Introduction

Reaching movements are governed by estimates of sensory and environmental quantities. If performed under uncertainty they are often based on prior expectations that summarise previous relevant information. We study the temporal evolution of the decision priors in a two-alternative forced-choice movement task.

## Methods

We acquired data from four right-handed and four left-handed participants who performed an obstacle avoidance task using a manipulandum (Phantom 3.0). In each trial, downward movements were initially directed towards an intermediate target. When arriving at the via-point the location of the final target was revealed which indicated whether the obstacle was to be circumvented on its right or left side. The task was spatially symmetric and the laterality of the final target was overall balanced, the first tens of trial, however, were deliberately weakly biased towards one side. The validity of a trial required reaching the final target within a short time interval by passing the obstacle on the correct side. When departing from the intermediate target, participants often moved briefly towards the wrong side before reverting this preliminary decision and proceeding toward the final target along the correct side. The interim movements were extracted from four blocks of 250 valid trials each for both dominant and non-dominant hand for each participant. The data were statistically analysed using linear models for a local description of the time series of interim movements and a non-linear model for the description of the dynamics of the decision priors.

## Results

Analysing directional decisions in dependence on a bias in target presentation, we find that all participants have a strong tendency to adopt the initial bias from the presented distribution. This tendency is strengthened during breaks between sessions. Only when a large number of trials that provide evidence to the contrary a small but significant adjustment of the decision strategy is observable. The results are reproduced independently of handedness. After a large number of strongly biased interim movements the subjects tend to realise the suboptimality of their decision strategy and return briefly to a less biased behaviour which is, however, typically unstable. These phenomena are shown to be independent of handedness.

Linear modelling (SARMA) revealed that the movement decision becomes soon nearly independent of the task, but is predictable by earlier movement decisions although information from earlier trials is statistically irrelevant. This effect can be captured for all naïve participants by a non-linear model of symmetry breaking in the decision task. Instead a nonlinear model of decision making is required which involves different time scales for formation of estimates and movement generation.

## Conclusion

Instead of constructing an internal representation of the statistical distribution of the task and their sensory uncertainty, the participants were forming a reflexive prior of their previous actions, while the performance of the task was achieved by reactive control, cf. [1]. Although information from earlier trials is statistically irrelevant, its effect could not be ignored and introduced a strongly biased perception of the task. We conclude that our results do not question the Bayesian paradigm in sensorimotor control, but may introduce an inaccurate and potentially suboptimal representation of the environment.
